# Meiotic chromosome mobility in fission yeast is resistant to environmental stress

**DOI:** 10.1038/srep24222

**Published:** 2016-04-14

**Authors:** Doris Illner, Alexander Lorenz, Harry Scherthan

**Affiliations:** 1 Institut für Radiobiologie der Bundeswehr in Verbindung mit der Universität Ulm, Neuherbergstr. 11, D-80937 München, Germany; 2 Institute of Medical Sciences (IMS), University of Aberdeen, Foresterhill, Aberdeen AB25 2ZD, United Kingdom

## Abstract

The formation of healthy gametes requires pairing of homologous chromosomes (homologs) as a prerequisite for their correct segregation during meiosis. Initially, homolog alignment is promoted by meiotic chromosome movements feeding into intimate homolog pairing by homologous recombination and/or synaptonemal complex formation. Meiotic chromosome movements in the fission yeast, *Schizosaccharomyces pombe*, depend on astral microtubule dynamics that drag the nucleus through the zygote; known as horsetail movement. The response of microtubule-led meiotic chromosome movements to environmental stresses such as ionizing irradiation (IR) and associated reactive oxygen species (ROS) is not known. Here, we show that, in contrast to budding yeast, the horsetail movement is largely radiation-resistant, which is likely mediated by a potent antioxidant defense. IR exposure of sporulating *S. pombe* cells induced misrepair and irreparable DNA double strand breaks causing chromosome fragmentation, missegregation and gamete death. Comparing radiation outcome in fission and budding yeast, and studying meiosis with poisoned microtubules indicates that the increased gamete death after IR is innate to fission yeast. Inhibition of meiotic chromosome mobility in the face of IR failed to influence the course of DSB repair, indicating that paralysis of meiotic chromosome mobility in a genotoxic environment is not a universal response among species.

Exposure to ionizing radiation (IR) induces a plethora of physico-chemical effects in irradiated cells including DNA damage^1^,^2^. Particularly DNA double strand breaks (DSBs) threaten the genomic stability of a cell and its survival. DNA misrepair can lead to mutations and missegregation of chromosomes that compromise the fitness of gametes as reflected by reduced sporulation after IR exposure of meiotic cells^3^,^4^. Furthermore, gamete production in mammals and humans is sensitive to environmental stressors like IR and reactive oxygen species (ROS); and elevated ROS levels have been noted in neurodegenerative diseases where they to affect the microtubule cytoskeleton^5^.

Meiosis halves the genome to compensate its doubling at fertilization. To this end, two successive rounds of chromosome segregation follow a single phase of DNA replication leading to the formation of haploid gametes or spores. Before homologous chromosomes (homologs) reductionally segregate, they pair lengthwise during the prophase of the first meiotic division. In most organisms homolog pairing relies on the formation of physiological DNA double strand breaks (DSBs) that are formed by the transesterase Spo11 during the leptonema substage of prophase I^6^. Preceding the intimate DSB-mediated homolog pairing, additional protein- or RNA-dependent mechanisms support the homolog recognition process (reviewed in^7^), as is the case in *C. elegans*^8^, mouse^9^ and *S. pombe*^10^. In many organisms intimate homolog pairing culminates in the formation of a synaptonemal complex. Ultimately, correct homolog segregation in the first meiotic division is ensured by at least one physical recombination-induced exchange per chromosome pair (reviewed in^11^).

Meiotic homology search in many organisms is associated with chromosome movements in the presence of physiological DSBs^12^,^13^,^14^,^15^. In the asynaptic meiosis of the fission yeast *Schizosaccharomyces pombe* a synaptonemal complex is absent but chromosomes are aligned by oscillating nuclear mobility driven by the astral microtubule-bound spindle pole body (SPB) to which telomeres are attached, giving rise to the so-called horsetail nucleus throughout much of prophase I^7^,^16^.

Meiotic chromosome mobility is thought to support the chromosome pairing process in meiotic prophase and is generally driven by cytoskeletal forces, either by microtubules (mammals, worms and fission yeast)^15^,^17^,^18^,^19^,^20^ or actin (budding yeast)^21^,^22^. Since it has been hypothesized that meiotic chromosome mobility may regulate adverse chromosomal interactions^22^,^23^,^24^, it may be speculated that the meiotic cell should seek to prevent illegitimate chromosome interactions in the presence of genotoxic DSBs seeded by genotoxins or ionizing radiation (IR). In line with this hypothesis it has been observed that low dose X irradiation of sporulating *S. cerevisiae* SK1 cells stalls meiotic chromosome mobility at a threshold dose of about 40 Gy being due to IR-induced radical stress and oxidation-induced collapse of the actin cytoskeleton^25^.

However, it is not known whether the IR- and ROS-induced stalling of meiotic chromosome movements in budding yeast relates to a protective mechanism that paralyzes chromosome mobility upon exposure to a genotoxic environment, or whether this effect solely relates to the sensitivity of the actin cytoskeleton to oxidative agents^26^, or an overall ROS sensitivity of this species. Thus, we asked the question how tubulin-driven chromosome mobility is affected by irradiation or ROS exposure. Since meiosis of the fission yeast *Schizosaccharomyces pombe* presents with a meiotic prophase that uses astral microtubule (MT)-driven meiotic chromosome mobility^12^,^18^, we used this model system for a comparison of IR effects on meiotic chromosome mobility with the actin-driven system of *S. cerevisiae*. To be able to follow chromosome movements in live meiotic *S. pombe* cells we employed strains that carry GFP-tagged tubulin (Atb2^27^) and Rec8, a subunit of cohesin, the latter being specifically expressed in meiotic prophase thus allowing a precise staging and recording of meiotic cells^28^,^29^.

To study the consequences of IR exposure on meiotic chromosome mobility we followed nuclear and chromosome movements in X-irradiated and non-irradiated live *S. pombe* prophase I cells expressing Rec8-GFP and Atb2-GFP for staging. Furthermore, we studied IR-induced ROS formation, the MT and actin cytoskeleton and spore viability in irradiated *S. pombe* cells undergoing meiosis in the presence or absence (MTs poisoned) of meiotic chromosome mobility.

## Results

### X irradiation reduces sporulation and spore viability

**Figure 1:**
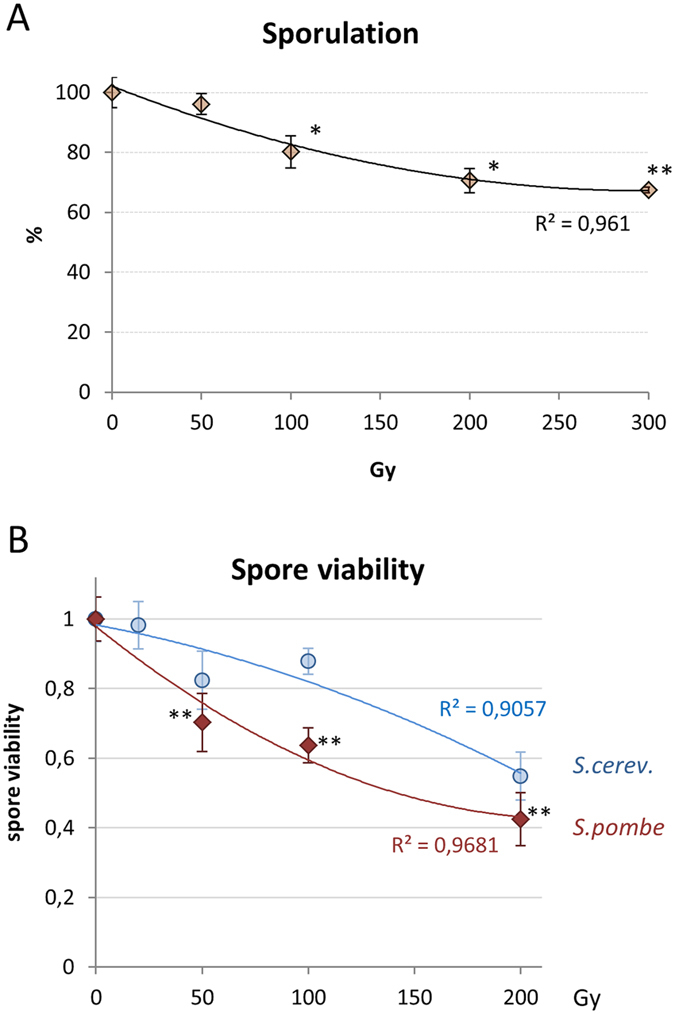
X irradiation reduces sporulation and spore viability. (**A**) Sporulation is significantly reduced after 100Gy (*p = 0.017), 200 Gy (*p = 0.0027) and 300 Gy (**p = 0.0008) relative to control. Data points reflect the mean of 3 experiments (±SD). (**B**) Spore viability in azygotic *S. pombe* and *S. cerevisiae* SK1 strains irradiated with 240 kV X rays. The differences between the respective doses analyzed in budding and fission yeast are highly significant (p < 0.0001). Data points reflect the mean of 4 technical repeats (±SD).

To determine the reaction of meiotic fission yeast cells to the exposure with IR we irradiated sporulating cultures with increasing doses of 240 kV X rays. Cells were irradiated 2–3 h after transfer to sporulation medium when most cells are in the horsetail stage, sporulation rates were determined 20 h post IR in three independent experiments. The diploid strain used carries GFP-tagged versions of the meiotic cohesin subunit Rec8 and of alpha-tubulin Atb2, which allowed for simultaneously visualization of meiotic prophase nuclei and MTs. A significant (p = 0.017) dose-dependent reduction of sporulation was noted at doses ≥100 Gy (10 krad) resulting in a reduction to about 33% of control at 300 Gy (p = 0.0008) (Fig. 1A).

Since quality control of meiosis is linked to generation of viable progeny (spores)^30^ we next determined spore viability after irradiation in 6 technical replicates. Here, spore viability dropped with increasing doses to 42% of control after 300 Gy X-irradiation (Fig. 1B), indicating severe damage to *S. pombe* meiocytes and spores.

In *S. cerevisiae* SK1 meiosis sporulation is also reduced by IR, but already to 34% by 50 Gy X irradiation^25^, indicating a much more radiosensitive meiosis in this species. Intriguingly, the fewer spores that formed in *S. cerevisiae* at higher radiation doses displayed a higher relative spore viability than *S. pombe* spores irradiated at the same level; the differences being highly significant (p < 0.0001) (Fig. 1B).

It has been proposed that meiotic chromosome motility is involved in the regulation of meiotic DSB repair. To see whether this is the case for IR-induced DSBs in meiotic cells, we irradiated motile and Thiabendazole (TBZ)-paralyzed horsetail cells at concentrations that inhibit horsetail movements without affecting spore viability^19^ and determined sporulation and spore viability. We observed that the sporulation rate of motile and immotile irradiated meiocytes were similar in the presence or absence of irradiation (Suppl. Fig. S1), refuting a role of meiotic chromosome mobility in the regulation of the repair of (IR-induced) DSBs.

### Meiotic chromosome and nuclear motility in *S. pombe* is radio-resistant

With the onset of first meiotic prophase *S. pombe* chromosomes attach with their telomeres to the SPB that moves along bundles of MTs dragging the nucleus behind, giving rise to recurrent elongations of the nucleus during much of prophase I, which is known as the horsetail movements^12^,^19^. In our experiments nuclear oscillations and horsetail movements occurred as described earlier (Suppl. Video 1), with horsetail movements being sensitive to the MT drug TBZ.

**Figure 2:**
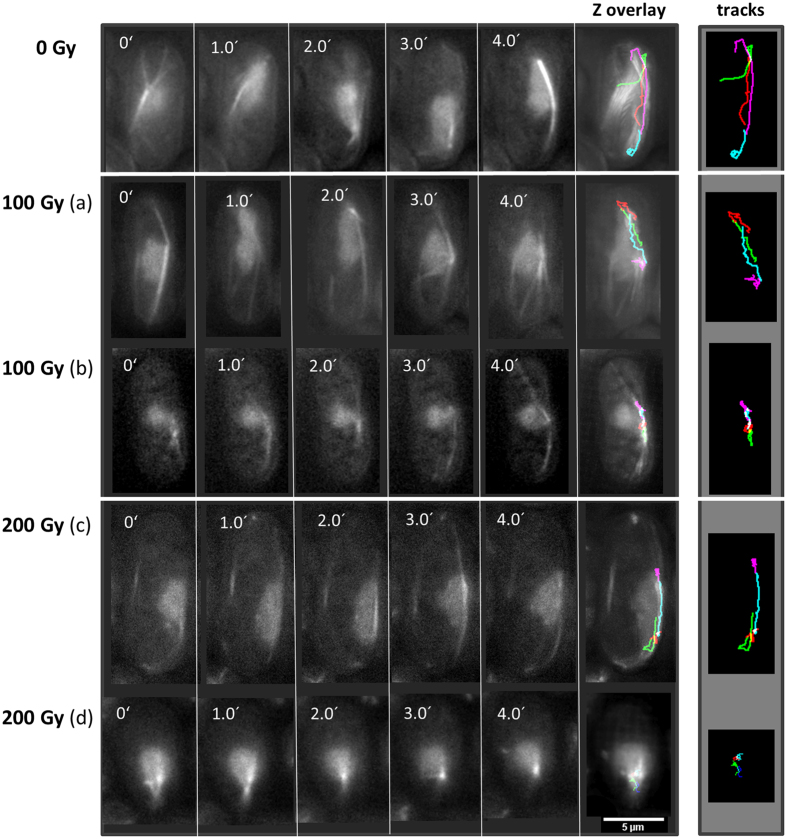
Image series of horsetail mobility under X irradiation. Without IR exposure (0 Gy; recording time 4 min) horsetail nuclei perform long continuous movements. Tracking of the leading edge of the nucleus (SPB) leads to continuous trails (each minute displayed in a different color) over the time recorded. Exposure to 100 Gy (**a**) X IR induced temporal slowing down of the mobility, resulting in alternating short and long tracks. In a subset of 100 Gy-irradiated nuclei (**b**) mobility is restricted to a small area and the nuclei display a condensed morphology. 200 Gy X IR (**c**) also induced alternating short and long tracks of horsetail mobility, while a subset of nuclei (**d**) displayed compete paralysis of mobility. Z overlays to the right show the tracks imposed on the cellular image, while “tracks” show the four differently colored 1 min tracks travelled by the leading edge only.

**Figure 3:**
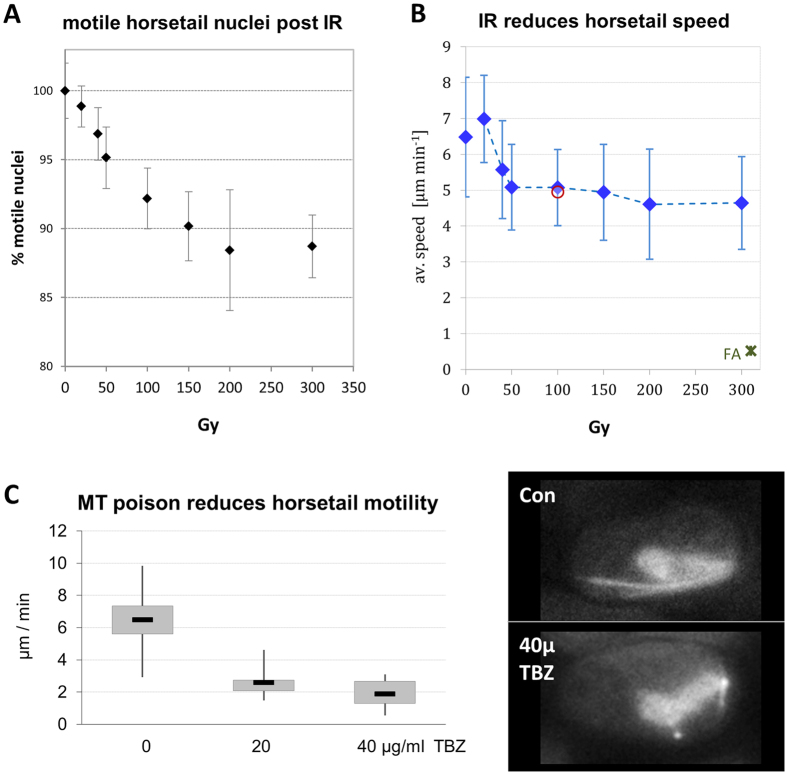
Dose-dependent reduction of horsetail mobility by X ray exposure. (**A**) Irradiation with doses of up to 200 Gy leads to a linear reduction (r^2^ = 0.937) of the number of mobile horsetail nuclei per sporulating culture. Beyond 200 Gy there was no further effect. Data points reflect the mean of 3 experiments (±SD). (**B**) X irradiation significantly reduces average horsetail speed at ≥50 Gy (p < 0.0001) relative to control, while there is only a slight insignificant further reduction up to 300 Gy, indicating that MT-driven horsetail mobility is radioresistant. Controls: The red circle indicates the horsetail speed of cells in sporulation medium without minerals and vitamins after irradiation with 100 Gy, to test for nutrient effects on speed measurements. The dark-green asterisk at the lower right (FA) indicates the mobility of the leading edge of the horsetail nuclei after 4% formaldehyde fixation. (**C**) Effect of the MT-poison TBZ on horsetail mobility. Treatment of sporulating culture with TBZ (20 and 40 μg/ml TBZ) lead to a highly significant (p < 0.001) reduction of horsetail speed. The images to the right display a control cell (Con; nuclear equator shown) and a cell treated with 40 μg/ml TBZ (40 μ TBZ) that exhibits the loss of long astral microtubules. Box blot, whiskers show minimum and maximum speed observed.

Irradiation of horsetail-stage cells with increasing doses of X rays of Rec8-GFP and Tubulin-GFP still revealed the typical mobility of meiotic nuclei driven by astral MT oscillations as revealed by time lapse cinematography (Fig. 2). However, tracks of the leading edge (SPB) of the nucleus, which in controls resulted from continuous long distance movements, were in X irradiated cells interdigitated with shorter tracks (periods) of slow mobility or paralysis (Fig. 2). An increasing number of irradiated cells displayed paralyzed horsetail mobility peaking at 12% of immobile horsetail nuclei at 200 Gy (Fig. 3A). Quantitative image analysis of the movements of the leading edge (the SPB) of horsetail nuclei revealed an average speed of 6.2 μm/min (±1.81 SD) (Fig. 3). Irradiation with 50 Gy X rays led to a significant (p < 0.001) reduction to 5.2 μm/min (±1.34 SD), while there was only a weak insignificant (p = 0.138) further reduction at higher doses to an average horsetail speed of 4.6 μm/min (±1.29 SD) at 300 Gy (Fig. 3B). Obviously, *S. pombe* cells keep moving meiotic chromosomes even in the presence of high doses of IR, which is likely dependent on a potent antioxidant response^31^ that is able to compensate IR-induced radical flux at a dose rate of 3 Gy/min (a higher dose rate was precluded for technical reasons), indicated by the absence of a further reduction of horsetail speed at doses >100 Gy (Fig. 3B) and the absence of a further increase in paralyzed cells at doses ≥200 Gy (Fig. 3A).

Controls with the MT-drug TBZ prior to irradiation reduced horsetail motility to an average of ~2 μm/min (Fig. 3C), while fixation with 4% formaldehyde abrogated nuclear mobility and left only limited local oscillations at 0.52 μm/min (Fig. 3B), likely owing to temperature-driven Brownian movements.

Because antioxidants can ameliorate radical effects on meiotic chromosome mobility^25^, and since *S. pombe* sporulation medium contains vitamins, we also performed controls with sporulation medium lacking vitamins and minerals and determined the horsetail speed at 100 Gy, but this rendered similar results to the standard conditions (Fig. 3B), excluding any scavenging activities of sporulation medium components.

### ROS mediate the reduction of bivalent mobility

**Figure 4:**
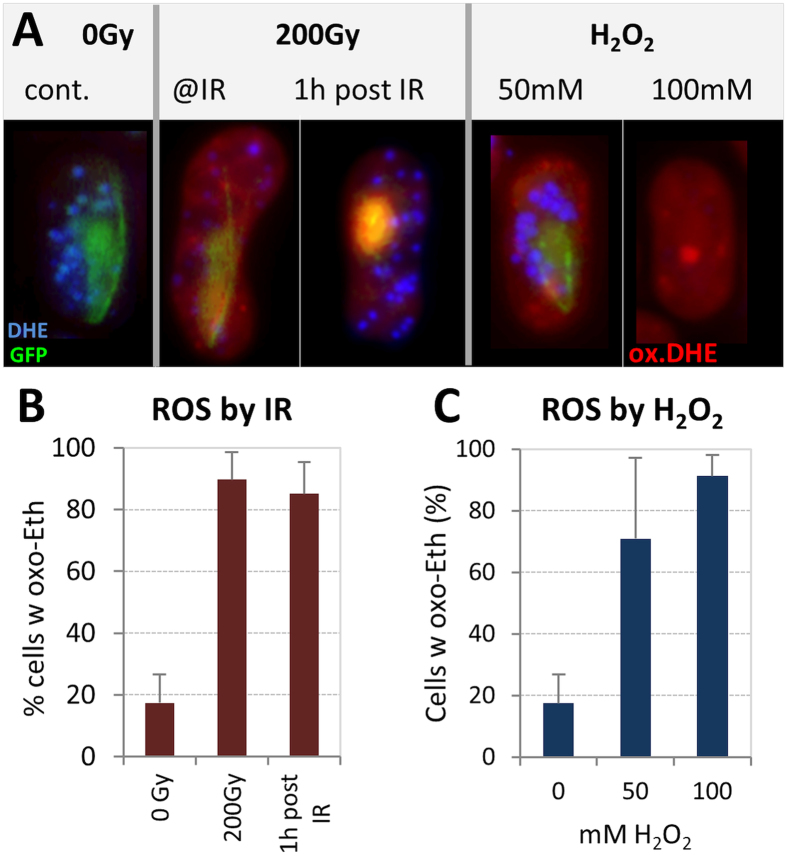
Analysis of the formation of reactive oxygen species (ROS) in irradiated Rec8-GFP expressing horsetail cells by di-hydroethidium (DHE, blue) staining. ROS exposure converts DHE to oxidized ethidium (oxo-Eth, red). Image recording was normalized by addition of fluorescent TetraSpecks. (**A**) Control cell (0 Gy) with a Rec8-GFP tagged (green) nucleus showing blue reduced DHE accumulations in the cell. 200 Gy: X irradiated cell displaying oxidized DHE (ethidium, ox.DHE; red) in the entire cell, while the nucleus appears reddish due to ethidium binding to DNA. Cell 1 h post IR still showing slight red cytoplasmic labelling, while the nucleus appears orange due to colocalization of green Rec8-GFP and red Ethidium (ox.DHE). Still, non-oxidized blue DHE aggregates can be seen. H_2_O_2_: Cells treated with H_2_O_2_ showing cytoplasmic red ROS labelling and a green horsetail nucleus at 50 mM H_2_O_2_, while 100 mM H_2_O_2_ quenched any endogenous fluorescence except for the red ethidium (ox.DHE) label. The red dot represents an unknown ethidium-affine structure in the cytoplasm. For a split channel display of the images see Suppl. Fig. S2. (**B**) Irradiation induces persistent ROS in 90% of 200 Gy-irradiated cells. (**C**) H_2_O_2_-induced ROS – a dose of 100 mM renders values similar to 200 Gy X irradiation.

Ionizing radiation creates radicals and reactive oxygen species that in budding yeast have been shown to reduce actin-dependent meiotic chromosome mobility^25^. Thus, we determined whether IR induces ROS in fission yeast cells by loading sporulating cells with the blue ROS probe dihydroethidium (DHE). Irradiation of DHE-containing cells with 200 Gy disclosed the oxidized red form of DHE (ox-DHE/Ethidium) in 90% of horsetail cells directly after IR exposure (Fig. 4). One hour post IR 85% of horsetail nuclei still exhibited red ethidium fluorescence (Fig. 4A,B; Suppl. Fig. 2), indicating that IR exposure induced lasting cellular ROS formation, like in budding yeast^25^. To compare this with chemically-induced ROS, we next treated cells with increasing concentrations of H_2_O_2_. In our hands, 50 mM hydrogen peroxide induced ROS in 70% of cells, while 100 mM H_2_O_2_ completely oxidized all cellular DHE indicating ROS induction in 91% of cells (Fig. 4A,C), which mirrors the situation observed after 200 Gy X-IR (Fig. 4).

### Hydrogen peroxide-induced ROS paralyze horsetail motility

**Figure 5:**
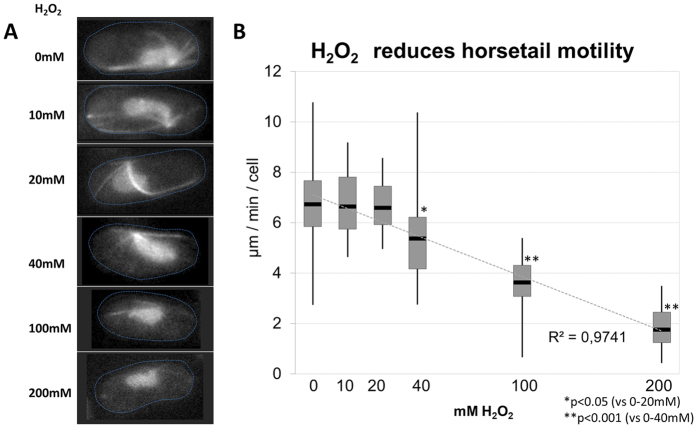
Effects of H_2_O_2_ treatment on horsetail mobility. (**A**) Images showing the typical appearance of horsetail cells after exposure to increasing concentrations of H_2_O_2_ in sporulation medium. At doses >20 mM H_2_O_2_ Rec8- and alpha tubulin-GFP fluorescence is quenched. (**B**) Above this dose the average speed of the horsetail mobility is significantly reduced, likely by oxidative damage to microtubules and other cellular proteins. The reduction of horsetail motility by 200 mM H_2_O_2_ is similar to values observed after treatment with the microtubule drug TBZ (see Fig. 3C). ROS induced with 100 mM H_2_O_2_ render horsetail speed values similar to 200 Gy X irradiation.

Inducing ROS with increasing concentrations of H_2_O_2_ revealed that *S. pombe* meiocytes display wild-type-like chromosome mobility in the presence of up to 20 mM H_2_O_2_ (Fig. 5; p = 0.756). Intriguingly, this dose is well above the doses used to activate the stress response in *S. pombe*^32^,^33^ and completely paralyse *S. cerevisiae* meiotic chromosome mobility^25^. At higher H_2_O_2_ concentrations the average horsetail motility was significantly reduced to 5.4 μm/min at 40mM (p = 0.012) and to below 2 μm/min at 200 mM (p < 0.0001). Corresponding effects for the horsetail speed-reduction were noted for 40 mM H_2_O_2_ treatment (Fig. 5) and the 50 Gy-induced reduction to 5 μm/min (Fig. 3B). The inhibitory ROS effect on meiotic chromosome mobility was confirmed by feeding the meiocytes the radical scavenger NAC (N-acetyl-L-cysteine) prior to IR treatment. NAC did ameliorate the IR-induced reduction of chromosome mobility leading to a 1.12-fold improvement on average in horsetail motility relative to cells without radical scavenger (Suppl. Fig. S3).

While elevated ROS levels have been noted to affect the microtubule cytoskeleton in neurodegenerative diseases (e.g.^5^), the data obtained in fission yeast meiocytes suggest a significant ROS resistance of the astral MT-driven horsetail movements, which contrasts with the ROS sensitive, actin-driven meiotic chromosome motility of budding yeast^25^. The difference likely relating to a potent antioxidant response in fission yeast^34^.

To exclude potential differences on the transcriptional level to the SK1 budding yeast strain studied previously, we investigated the expression of antioxidant response genes during sporulation of *S. cerevisiae* using the SK1 meiotic transcriptome at the Germonline database^35^. All tested antioxidant response genes like superoxide dismutase, catalase, glutathione peroxidases and thioredoxins showed strong expression during sporulation and vegetative growth (Table 1) of the *S. cerevisiae* SK1 strain^36^. Furthermore, manganese complexes have been found to be potent antioxidants in radioresistant prokaryotes and budding yeast^37^. Like for the enzymes noted above, all relevant Mn-antioxidant genes tested (ATX2, BSD2, CCC1, PHO80/85 and SCH9) showed high expression in SK1 sporulation. These data exclude potential gene-specific effects. Still, posttranscriptional differences or yet unknown antioxidant activities/regulatory circuits may contribute to a more potent antioxidant response in fission yeast.

### X irradiation induces genotoxic DSBs in meiotic prophase cells

**Figure 6:**
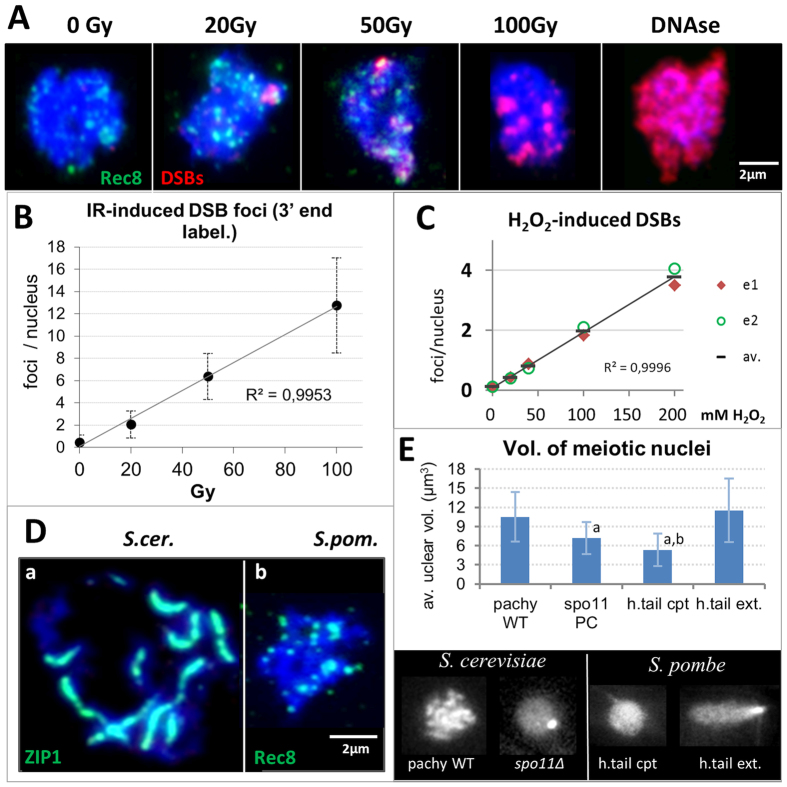
Detection of IR-induced DSBs by 3′-end-labelling of surface-spread horsetail nuclei. (**A**) Maximum projection images showing spread nuclei stained for DSBs by 3′-end-labelling (Cy3-dCTP, red). Horsetail nuclei are tagged with Rec8-GFP dots (green); DSB signals (red); DNA is stained with DAPI (blue). Positive control: DNAse I-treated cells (DNAse) show an overall reddish labelling. Scale bar: 2 μm. (**B**) Graph showing the average frequency of irradiation-induced DSB foci in spread horsetail cells identified by punctate Rec8-GFP signals. Background labelling in non-irradiated controls was 0.46 foci/cell. There is a dose-dependent linear increase (R^2^ = 0.9953) of IR-induced DSBs at 1.3 DSBs per 10 Gy. (**C**) Average frequency of DSB foci in H_2_O_2_-treated horsetail cells. Background labelling in non-exposed controls was 0.11 foci/cell. There is a dose-dependent linear increase of H_2_O_2_-induced DSBs with 2 DSBs at 100 mM. The average values (av.) are from 2 independent experiments as shown (e1, e2 = experiment 1 and 2). (**D**) Nuclear spreading of meiocytes leads to much wider chromatin distribution with *S. cerevisiae* pachytene cells (Zip1-GFP-tagged; see^25^) than with *S. pombe* Rec8-GFP expressing horsetail meiocytes, suggesting a different chromatin organization/state in *S. pombe* nuclei (DNA, blue). Bar: 2 μm. (**E**) Graph showing average nuclear volume of fission and budding meiocyte nuclei (±SD) as revealed by image analysis of Rec8-GFP (*S. pombe*) and Zip1-GFP (*S. cerevisiae*) expressing live cells. While pachytene nuclei and extended horsetail-stage nuclei have a similar volume, the volume is significantly smaller in *spo11*∆ budding yeast nuclei (68% of wt; p<0.001) that do not form an SC. The rounded *S. pombe* horsetail-stage nuclei are even smaller in volume (46% of extended horsetail nuclei, or 51% of wild-type pachytene); ^a^p < 0.001 relative to pachytene and ext. horsetail nuclei, ^b^significantly smaller than pachytene (p < 0.0001), *spo11*∆ (p = 0.01) and ext. horsetail nuclei (p < 0.0001). Below: representative images of a *S. cerevisiae* pachytene (pachy) and a polycomplex (PC, white dot, used as stage marker) expressing *spo11*∆ SK1 meiocyte nucleus. *S. pombe:* prophase I nuclei expressing Rec8-GFP in rounded (h.tail cpt; the SPB passes the trailing edge on its way to the other cell tip) and extended horsetail-stage nuclei (h.tail ext.; SPB at the cell tip). Gray scale images, bar: 2 μm.

The reduced spore viability observed above could be the consequence of genotoxic DSBs. To determine the amount of IR-induced DSBs in meiotic fission yeast cells, we stained DSBs with a modified 3′-end labelling protocol that reveals IR-induced DSBs in meiotic cells but fails to detect physiological meiotic DSBs^25^ as indicated by nearly identical focus numbers in irradiated wild-type and *spo11∆* budding yeast meiocytes that fail to form physiological DSBs^38^. Spreads of non-irradiated meiotic prophase fission yeast cells (identified by Rec8-GFP expression) displayed a background level of on average 0.46 DSB foci/cell (Fig. 6A,B). Irradiated meiotic prophase cells displayed an average of 13 DSB foci after 100Gy exposure, with the dose response being linear at an average of 0.13 foci/Gy/cell (Fig. 6B). In contrast, H_2_O_2_ treatment induced only 4 DSBs at the highest concentration used (200 mM; Fig. 6C) indicating that the ROS-induced effects are not DSB-dependent.

The obtained yield of IR-induced DSB foci is somewhat below the 0.15 DSB/Gy expected in G_2_-M-phase cells^39^, which may relate to methodological differences. Additionally, this may also relate to the high activity of DNA repair by homologous recombination in meiotic *S. pombe* cells and to its stronger antioxidant defense.

### Differences in the meiotic chromosome scaffold influence nuclear compaction

Using 3’-end labelling, we noted a different DSB dose response of meiotic budding yeast cells compared to fission yeast cells, the former displaying 0.2 DSB foci/Gy^25^ versus 0.12 DSB/Gy in fission yeast, at the same radiation quality and dose. However, both yeasts display similar genome sizes (14.1 Mb for *S. pombe*^40^; 12.1 Mb for *S. cerevisiae*^41^), while *S. pombe* lacks a synaptonemal complex (SC) that enforces intimate homolog pairing^42^,^43^. To see whether differences in the meiotic chromosome scaffold (linear elements in *S. pombe*^43^,^44^, SC in *S. cerevisiae*^45^,^46^) affect the preparations obtained for DSB analysis, we inspected nuclear spreads of the two species and noted that *S. cerevisiae* pachytene nuclei spread over a much wider area than the *S. pombe* meiotic prophase nuclei (Fig. 6D), likely because individual SC-connected budding yeast bivalents separate from each other more easily during nuclear spreading, while the nuclear organization with numerous linear elements may render the fission yeast meiotic prophase nuclear chromatin more compacted after spreading. To further explore the latter possibility, we determined the nuclear volume in live Rec8-GFP expressing *S. pombe* prophase cells and in Zip1-GFP expressing *S. cerevisiae* pachytene cells. Volume reconstruction from 3D image stacks of Hoechst-stained live meiocytes indicated that fission yeast nuclei may be smaller. Since 3D measurements based on Hoechst staining may be prone to artifacts due to the flaring of the dye, we next calculated the volume of prophase I nuclei from images of movies of live GFP-expressing meiocytes of both species (Fig. 6E). During meiotic prophase *S. pombe* nuclei undergo oscillating movements, changing between an extended horsetail shape when the SPB reaches the cell tip (Suppl. Movie 1) and a rounded compact form when the SPB is returning to the bulk of the chromosomes in the cell center^18^. Thus, we determined the nuclear volume in extended and rounded forms of prophase nuclei. We also included measurements of wild-type pachytene and *spo11*∆ pachytene-like nuclei from *S. cerevisiae* to determine differences in nuclear organization between these two species; *spo11*∆ does not form SC but a polycomplex^47^ that is seen as a nuclear dot that we used as a pachytene-like stage marker. Our volume analysis revealed that rounded horsetail-stage nuclei in the center of *S. pombe* meiocytes were significantly smaller in volume than fully extended horsetail nuclei (p < 0.0001). While the latter was similar in volume to wild-type *S. cerevisiae* pachytene nuclei, *spo11*∆ nuclei were reduced in volume relative to wild-type ones (p = 0.01) and extended horsetail nuclei of fission yeast (p < 0.0001; Fig. 6E). Rounded *S. pombe* horsetail-stage nuclei were even smaller than *S. cerevisiae spo11*∆ nuclei that lack a rigid SC (Fig. 6E). These results indicate that differences in the meiotic chromosome scaffold influence nuclear organization and in turn the behavior of the chromatin during nuclear spreading. It thus seems likely that technical reasons, i.e. more limited spreading of *S. pombe* horsetail nuclei, may have contributed to the lower estimate of IR-induced DSB numbers in fission yeast.

### Homologue pairing and cell cycle progression under irradiation

**Figure 7:**
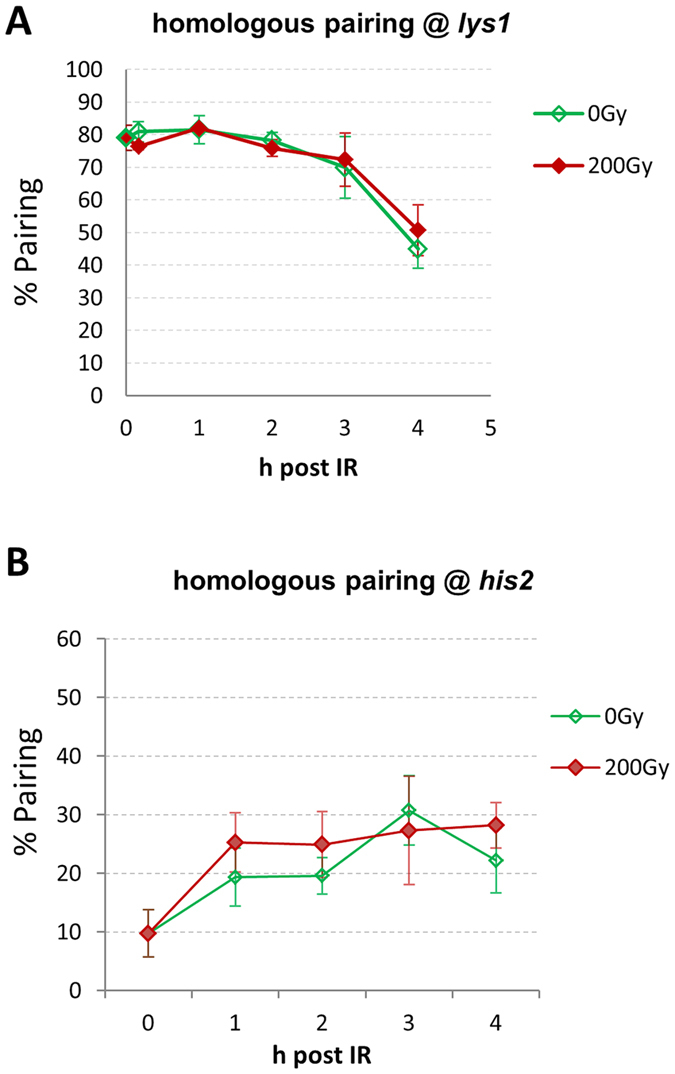
Homologous chromosome pairing is not affected by IR. (**A**) Pairing of LacI-GFP dots at *lys1* (close to centromere 1) is similar in control (0 Gy) and 200 Gy X-irradiated horsetail cells 3 h post induction. (**B**) The pairing at *his2* (interstitial region on chromosome 2) was slightly but insignificantly faster in the first hour post IR but remained on a similar level to control (0 Gy) between 1 h and 4 h after irradiation with 200 Gy 1 h post induction. The differences being insignificant.

Next we determined homologous chromosome pairing at two different LacO/LacI-tagged loci. First we determined pairing at a centromere-near locus (*lys1*) on the right arm of chromosome I^48^,^49^. When sporulating cells were irradiated with 200 Gy in the horsetail stage, high levels of pairing at the centromere region were similar to the unirradiated control (Fig. 7) and agree with previous findings^16^. Pairing at an interstitial arm-region on chromosome 2 (*his2*)^50^ was more dynamic and slightly but insignificantly faster in the first hours post IR. Similar pairing levels persisted for up to 4 h post IR (1 h post induction), but less so in the control (Fig. 7) with the differences being insignificant (p = 0.405).

**Figure 8:**
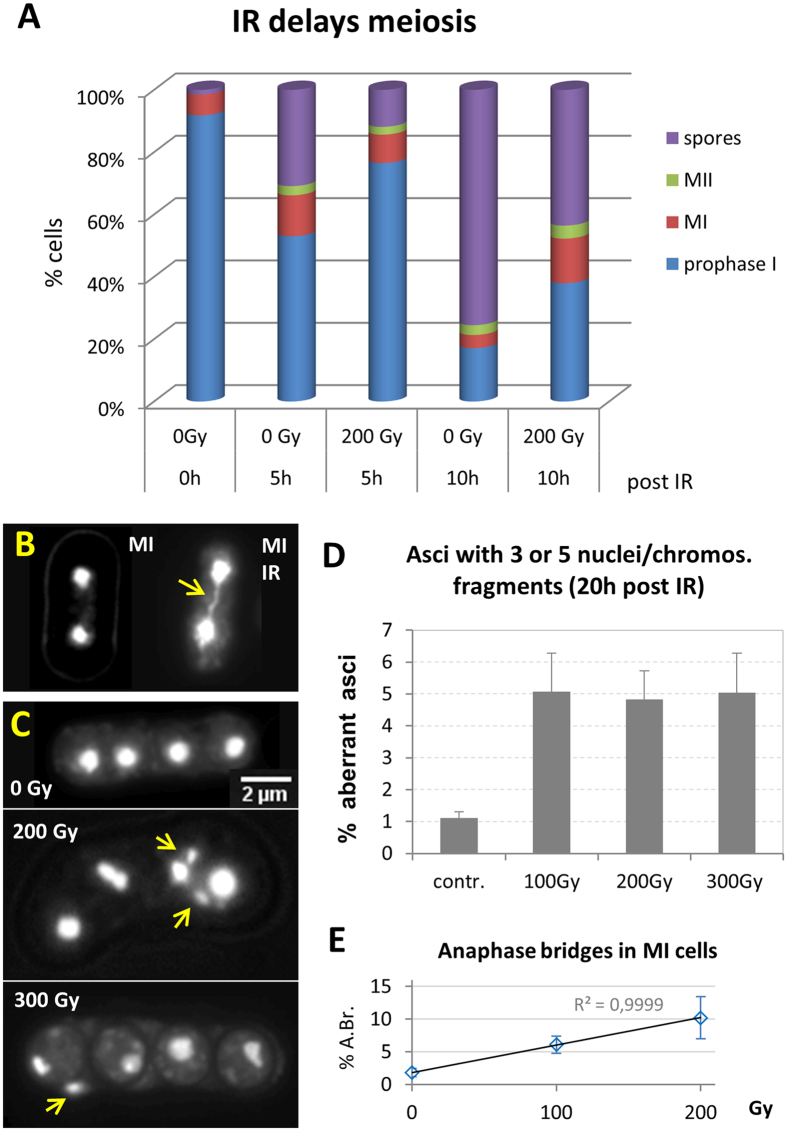
Metaphase progression is altered by IR. (**A**) Proportion of Metaphase I (MI) and II (MII) cells and spores as determined by DAPI staining in sporulating cultures. Before irradiation (0 Gy, 0 h) 92% of cells were in prophase I and 6.7% of cells were in MI. Five h thereafter (0 Gy, 5 h), there were 53% prophase I cells, 13% MI, and 31% ascospores. Irradiation delayed prophase I progression in that 77% of cells still were in first meiotic prophase 5 h post IR (200 Gy, 5 h), as identified by horsetail morphology and Rec8-GFP expression. This was only the case for 53% of control cells. At 10 h post IR 38% of irradiated cells were still in prophase I, while this was only seen for 17% in the control (0 Gy, 10 h) where 74% of cells had formed spores. (**B**) DAPI image of anaphase I cells. The control (left) shows two nuclei (DNA: white) and the 200 Gy X-irradiated cell (right) displays a chromatin stretch between the daughter nuclei (arrow) indicative of an anaphase bridge likely caused by a dicentric chromosome, since the frequency of these cells increases with dose (below). (**C**) Ascospores without (0 Gy) and after irradiation. The 200 Gy ascospore below exhibits more than 4 nuclei, with the excess nuclei being smaller (arrows), being indicative of anaphase-lagging of acentric chromosome fragments due to unrepaired DSBs. The 300 Gy ascospore displays one chromosome fragment (arrow) outside the spore. (**D**) Frequency of asci with aberrant numbers of nuclei/chromosome fragments. Irradiation with 100–300 Gy X rays induced an average of 5% asci that displayed chromosomal fragments. (**E**) Percentage of Metaphase I (MI) cells with anaphase chromatin bridges (A.Br.; see B) without IR (n = 380 MIs analyzed) and after exposure to 100 Gy (n = 325) and 200 Gy X rays (n = 284). There is a linear increase with dose of about 2% anaphase bridges/50 Gy. Cells of three independent experiments were analyzed 5 h post IR; average ± SD shown.

To see whether these results may be related to a delay in meiotic progression we monitored the appearance of metaphase I and II cells in control and 200 Gy-irradiated sporulating cultures. At the time point of irradiation 92% of cells were in prophase I. Five hours post IR 76% of exposed cells were still in prophase I, as identified by horsetail morphology and Rec8-GFP expression, while in the control only 53% of cells were still in prophase I (Fig. 8A). At this time point 13.2% of control cells were engaged in the MI division as indicated by binucleated cells (Fig. 8B), while in the irradiated aliquot only 9% of cells had reached the MI division. At 10 hours post IR 14.3% of irradiated and 4% of control cells still were in the MI division (Fig. 8A). At this time point 74% of control cells but only 43% of irradiated cells had formed spores. This indicates that irradiation slows down prophase I progression with irradiated cells reaching metaphase I with a delay. They also seem to spend more time in the latter stage, as indicated by ~14% MI cells 10 h post IR, which may relate to the presence of still unrepaired or misrepaired DSBs.

IR-induced misrepair of DSBs generates dicentric chromosomes (carrying two centromeres), and acentric fragment(s) that are also seen after failure of DSBs repair. To see whether this is the case, we inspected nuclear integrity in DAPI-stained preparations. Indeed we observed anaphase bridges and chromosome fragments in irradiated cells (Fig. 8B). Investigation of the frequency of IR-induced anaphase bridges yielded 1.8% binucleated MI cells with chromatin bridges in control cultures, while there was a linear dose-related increase at approximately 2% anaphase bridges per 50 Gy (Fig. 8E), mirroring the observed linear increase of IR-induced DSBs. DAPI-bright chromosome fragments indicative of acentric chromosome fragments were often excluded from spores in asci of irradiated cells (Fig. 8C). A 5-fold increase of aberrant asci in irradiated cells (Fig. 8D) suggests that irradiation induced chromosome missegregation and fragmentation by faulty or absence of DSB repair.

### IR impairs the actin cytoskeleton in horsetail nuclei and spores

Meiotic chromosome movements in budding yeast meiosis depend on actin polymerization^21^,^22^. In *S. pombe* meiosis the actin cytoskeleton is largely peripheral and contributes to cell growth and forespore membrane formation^51^,^52^.

**Figure 9:**
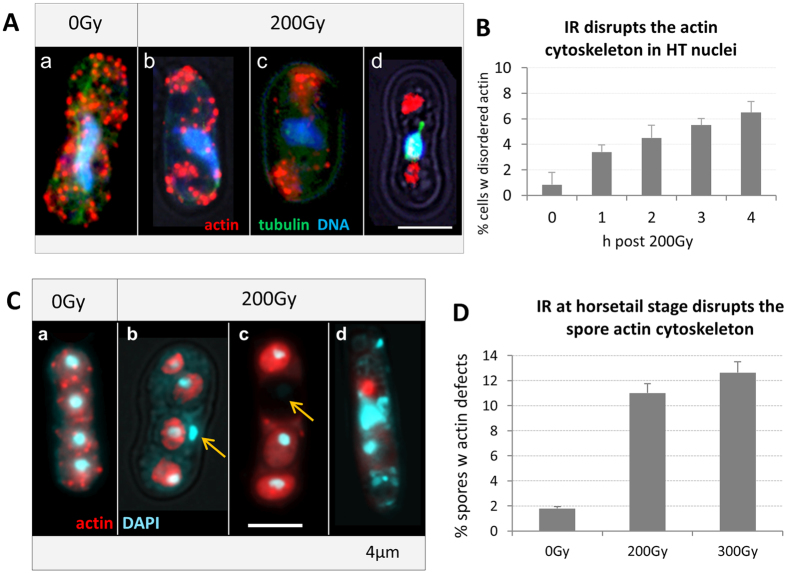
The actin cytoskeleton of horsetail cells. Actin is stained in red, tubulin in green, DNA in blue (DAPI). Maximum projections of image stacks shown. (**A**, a) Non-irradiated horsetail cell displaying a normal actin cytoskeleton characterized by numerous cortical patches that are of similar size and distributed throughout the cell. Irradiation with 200 Gy leads to an aggregation of the actin patches (**A**, b–d). (**B**) The percentage of cells with disordered actin distribution increases with time after 200 Gy X IR. (**C**) Actin (red) and DNA (blue) distribution in control (a) and ascospores formed after 200 Gy irradiation of horsetail cells (b–d). (b) IR-induced DNA fragmentation leads to DAPI bodies (light blue) outside spores (arrow). (c) The actin cytoskeleton fails to form patches and is sometimes absent in spores that lack DNA (arrow). (d) Ascus showing severely damaged nuclei, spores and precipitated actin (red dot). Bar: 4 μm. (**D**) Irradiation in the horsetail stage induces defective ascospores. Percentage of ascospores investigated (>150 from 3 different experiments; ±SD).

When we investigated the actin cytoskeleton in control and irradiated horsetail cells by TRITC-Phalloidin staining we noted the expected actin patches in control prophase cells (Fig. 9A), corroborating previous reports^51^,^52^. At doses ≥200 Gy there was a disordered actin distribution and the formation of actin bodies that are indicative of actin oxidation^53^. In the control, actin patches were scattered cortically around the cell, while IR induced the aberrant agglomeration of actin patches in about 6.5% of all horsetail nuclei 4 hours post IR (Fig. 9A,B). Investigating the *S. pombe* actin cytoskeleton in spores 20 h post IR revealed a disordered actin cytoskeleton in 11% of spores post 200 Gy and 12.6% post 300 Gy; with the cells being characterized by absence of actin patches, diffuse actin staining or complete absence of actin from some ascospores (Fig. 9C). The latter often being correlated with the absence of DNA from a spore (Fig. 9C).

Overall, these data suggest that IR exposure leads to formation of ROS that disturb the actin cytoskeleton that is important for spore formation. However, the presence of a disturbed actin cytoskeleton in only a little more than 10% of cells again indicates the presence of a sturdy antioxidant defense in *S. pombe* cells, rendering microtubule-driven horsetail mobility relatively radioresistant in comparison to the actin-driven meiotic chromosome mobility in *S. cerevisiae*.

## Discussion

To investigate the impact of ionizing radiation exposure on a microtubule-driven meiotic chromosome movement system, we exposed live *S. pombe* meiotic cells in the horsetail stage to increasing doses of X radiation. In haploid *S. pombe* strains 50% of cell killing (LD50) was achieved with 100 kV X irradiation at 215 Gy^54^. Irradiation of diploid prophase I cells in the horsetail stage with 300 Gy 240 kV X rays still allowed for 68% sporulation, indicating that sporulating diploid fission yeast cells are quite radioresistant. However, random spore analysis revealed that only 27% of spores post 300 Gy were viable, revealing an LD50 of about 150 Gy. The significant drop of spore viability relative to sporulation rate may relate to failure of repairing all IR-induced DSBs, as suggested by a dose-dependent increase of anaphase bridges in metaphase I cells and chromosome fragments in ascospores. Since such defects in spore formation were only seen in about 5% of asci and the viability of irradiated spores was below that of irradiated G1 phase cells^54^, it appears that additional factors such as quality control mechanisms blocking endolysis of asci with damaged spores^30^ and/or lax checkpoint control^55^ may also have contributed to the reduced spore viability observed. In contrast to this, sporulating *S. cerevisiae* is far more radiosensitive^25^, and spore formation is strongly reduced by irradiation, however, the few spores formed still displayed high spore viability (Fig. 1B). Whether the differences in spore viability after IR between the two model yeasts relates to variances in the checkpoints controlling quality of meiotic differentiation outcome remains to be determined.

To see whether meiotic chromosome mobility is influencing the repair of DSBs, we paralyzed mobile chromosomes with the MT drug TBZ. It appeared that absence of meiotic chromosome mobility did not improve the IR-induced reduction of sporulation or spore viability, indicating that chromosome mobility is not regulating the repair of (IR-induced) DSBs in prophase I of *S. pombe*.

It is known that one or two DSBs that remain unrepaired can kill a G1 yeast cell^56^. Investigation of IR-induced DSBs in spread meiotic prophase (horsetail) cells by a modified 3′-end labeling that labels only IR-induced genotoxic DSBs in meiotic cells^25^,^38^, revealed DSBs in *S. pombe* meiocytes at an average rate of 0.13 DSB/Gy with a linear dose response relationship. The obtained DSB yield in meiotic cells was similar to the 0.15 DSB/Gy induced in G_2_-M-phase *S. pombe* cells^39^. Since prophase I cells exhibit 4C DNA content, IR is expected to induce double the amount of DSBs relative to haploid G2 phase cells. However, compared to irradiated pachytene nuclei of budding yeast^25^ there was a somewhat lower DSB yield in our horsetail nuclei (0.13 vs 0.2 DSBs/Gy, respectively) exposed to the same radiation quality. This went along with a more limited surface-spreading of fission yeast meiotic prophase nuclei, which may be related to differences in the meiotic chromosome scaffold – in contrast to budding yeast, meiotic chromosomes of fission yeast lack a synaptonemal complex^43^,^44^, display recombination-independent homolog alignment and are maintained in a bouquet configuration throughout much of prophase I^12^,^42^,^57^. It thus seems possible that the *S. pombe* DSB/Gy values represent an underestimate, likely caused by label confluency of closely spaced DSBs in the more compact spreads of horsetail nuclei, thereby reducing the *in situ* detection of DSBs. Alternatively/additionally, a more potent antioxidant defense in *S. pombe* (see below) may have led to lower DSB numbers. Still, our DSB values are in the range of 0.15 DSBs/Gy observed in G_2_-M-phase *S. pombe* cells^39^ and the range of IR-induced DSBs in the germline cells of *C. elegans*^58^.

In all, it appears that the chromosome movement system of fission yeast is relatively insensitive to DSB formation, since most irradiated cells still performed robust movements in the presence of more than 20 IR-induced DSBs, which are only a fraction of the about 50–80 physiological DSBs per recombination-proficient *S. pombe* meiosis^59^,^60^.

In budding yeast meiotic chromosome mobility has been observed to be particularly vulnerable to IR exposure with mobility stalling in pachytene cells exposed to more than 40 Gy^25^. In contrast, after 50 Gy irradiation of *S. pombe* horsetail cells there was only a 21% reduction of the average speed by which horsetail nuclei traveled through the prophase I cell. This was seen over a dose range of 50–300 Gy, being in stark contrast to stalled mobility in >40 Gy irradiated *S. cerevisiae* pachytene cells. These differences seem to relate to the performance of the antioxidant protection systems in the two species seems. While *S. cerevisiae* SK1 meiocytes express the key components of the antioxidant defense response, IR >40 Gy still paralyzes their meiotic chromosome mobility due to actin oxidation. In contrast, only ~10% of irradiated *S. pombe* horsetail cells display damaged actin or tubulin cytoskeleton after irradiation with up to 300 Gy. Still, IR-induced ROS formation was detected by DHE oxidation and affected most cells directly after IR exposure in both yeasts.

While ROS affected MT- and actin-driven nuclear mobility to different extent, an additional protection against ROS could be achieved by pretreatment with the antioxidant NAC that improved horsetail speed approx. 1.2-fold in 50 Gy-irradiated horsetail cells, this value being similar to that obtained in antioxidant-treated budding yeast pachytene cells (1.3-fold protection^25^). Furthermore, high doses of H_2_O_2_ (200 mM) were required to dramatically reduce MT-driven horsetail motility, again pointing to a potent endogenous antioxidant defense in this species, especially, since similar effects were already achieved by 10 mM H_2_O_2_ in *S. cerevisiae* meiocytes.

The actin cytoskeleton has been found sensitive to ROS^53^, as is actin-dependent spore formation in both yeasts^51^,^52^. Here, we also noted perturbed forespore formation in irradiated postmeiotic cells, corroborating earlier analyses^51^. However, actin defects were only seen in about 10% of irradiated cells indicating that *S. pombe* is endogenously well protected against radical stress^31^,^61^. Efficient antioxidant protection of proteins by Mn-complexes have been noted in radioresistant prokaryotes and Mn-antioxidants are also used in yeast^37^. Future experiments will thus probe the radiation response of the chromosome moving system in meiocytes mutant for different components of the antioxidant systems.

## Methods

### Strains

*S. pombe* and *S. cerevisiae* strains used in this study are listed in Table 2. Strains were produced by standard genetic crossing procedures^62^.

In the original MS1428 strain *GFP-atb2*^+^ was marked with *kanMX*^27^ (Table 2). To make a *GFP-atb2*^+^ version marked with a clonNAT-resistance *cut12*^+^*-CFP*::*natMX* was crossed out. The resulting strain carrying only *GFP-atb2*^+^::*kanMX* was then transformed with a *natMX4* cassette (PCR product using oligonucleotides 5′-GTTTAGCTTGCCTCGTCCC-3′ and 5′-GATGGCGGCGTTAGTATCG-3′ and pAG25 as a template) employing a single-step marker switch protocol^63^,^64^,^65^. From this transformation clonNAT-resistant G418-sensitive colonies were selected, and the presence of *GFP-atb2*^+^ was verified microscopically.

All experiments were carried out in diploid strains undergoing azygotic meiosis upon starvation. *S. cerevisiae* Zip1-GFP strains (Table 2) were used as described earlier^21^,^25^.

### Cell culture and meiotic time-courses

Cell culture and meiotic time-courses were done as described in detail previously^25^,^66^,^67^. Synchrony of sporulation was controlled by live cell imaging at 2 and 3 hours after transfer to sporulation medium (PM-N). Experiments were continued when >70% of cells were expressing horsetail nuclei as determined by Rec8-GFP.

The extent of sporulation was assayed by DAPI staining of Ethanol-fixed cells. Spore viability was determined using established random spore analysis protocols for *S. pombe*^62^ and *S. cerevisiae*^68^.

### X irradiation

Five milliliters of a sporulating culture were irradiated in a slanted 50 ml Falcon tube at room temperature with 240 kV X rays at 13 mA (filtered with 3 mm beryllium) at a dose rate of 3 Gy/min using a fully shielded X ray device (Yxlon). The delivered dose was measured with a Duplex dosimeter (PTW) attached to the Falcon tube. Controls were sham irradiated.

### Drug treatment

H_2_O_2_: Aliquots (5 ml) of sporulating cultures were incubated in 10 mM, 20 mM, 40 mM, 100 mM and 200 mM H_2_O_2_ (Carl Roth) in sporulation medium for 20 minutes at 28 °C. Cells were pelleted by a brief spin, resuspended in sporulation medium and immediately subjected to live cell imaging.

The microtubule inhibitor Thiabendazole (TBZ; Sigma-Aldrich) was dissolved in DMSO at 50 mg/ml. TBZ (or DMSO alone) was added 30 min before analysis to sporulating cultures 3 h post induction, to result in 20 μg/ml or 40 μg/ml TBZ in sporulation medium. Cells were irradiated or sham irradiated, thereafter TBZ was washed out 3 times with sporulation medium 30 min after the end of irradiation to allow for repair initiation in the absence of motility.

Antioxidant treatment was done as described previously for budding yeast using N-acetyl-L-cysteine (NAC; Sigma-Aldrich)^25^. Sporulating cells were incubated for 40 min in sporulation medium containing 10 mM NAC. Thereafter, cells were irradiated with different radiation doses, or sham irradiated, and subjected to imaging or further experimentation.

### Immunofluorescent staining

The actin cytoskeleton was stained with phalloidin-TRITC (Sigma-Aldrich) as described^21^,^69^. Meiotic chromosome spreads were obtained according to published protocols^66^,^70^, immunostained with anti-GFP antibodies (Invitrogen, clone 3E6, 1/400) and washed 3 times 5 min each in PBS/0.5%Tween 20 (Sigma-Aldrich). Primary antibodies were detected with goat anti-mouse–Alexa488 secondary antibodies (1/500; Mobitec). More than 50 cells were counted per time-point. Experiments were done in triplicate.

### 3′ end-labeling of IR-induced DSBs

IR-induced DSBs were detected with a modified 3′end-labelling assay as described^25^. This assay detects unscheduled IR-induced DSBs only^38^. Trials using anti-Rad51 imunofluorescence, that also reveals physiological DSBs, failed to produce meaningful results (not shown). For end labelling, sporulating cells were transferred to wet ice and depleted from ATP by adding sodium azide (final conc. 0.04%) 10 min prior to IR to inhibit DNA repair. Directly after IR meiotic spreads were obtained as described^66^,^70^. Terminal deoxynucleotidyl transferase (TdT) labeling with Cy3-conjugated dCTP was carried out by covering H_2_O-rinsed spread preparations with 100 μl reaction buffer (NEB) containing 15U TdT enzyme (NEB), followed by incubation for 30 min at 37 °C and four 2 min washes in PBS. Slides were mounted in Vectashield containing DAPI as DNA counterstain (Vector Labs). 3D image stacks (step size 0.3 μm) were recorded using the ISIS image analysis system (MetaSystems) and converted to max projection images which were subjected to manual foci enumeration by an experienced investigator (DI).

### Detection of reactive oxygen species

ROS were detected in yeast cells with the ROS-specific probe dihydroethidium (DHE; Molecular Probes), a free radical sensor that in its reduced form exhibits blue fluorescence in the cytosol as described^25^. Oxidized DHE (ethidium) obtains a red fluorescence. Cells were incubated for 40 min with DHE (80 μM in sporulation medium) before IR. Thereafter, cells were washed once with sporulation medium, fixed for 10 min in 4% formaldehyde in PBS, washed once in PBS and embedded together with fluorescent 0.25 μm TetraSpecks (Invitrogen; diluted 1/1000) in antifade solution to normalize digital image recording. Cells that fluoresced red were scored as ROS-positive. Experiments were repeated at least twice.

### Live cell imaging and image analysis

Live cell imaging was done using our 4D live cell microscope system (TILL [now FEI]) as described in detail elsewhere^25^,^67^. Images (240 msec exposure time) were recorded every 2 seconds over 4 minutes. Longitudinal studies were done by recording several consecutive 4 min movies. Quantitative image analyses of time lapse movies were done using ImageJ (http://imagej.nih.gov/ij/). The plugin Manual Tracking (http://imagej.nih.gov/ij/plugins/track/track.html) was used for spot tracking and speed calculation. *S. cerevisiae* was analyzed as described previously^25^,^67^. Wide-field fluorescent images of IF-stained cells were recorded with the ISIS image analysis system (MetaSystems, Altlussheim) which also was used for computing maximum 3D image projections.

### DNA-staining and volume calculation

For volume determination live cells were stained with Hoechst 33342 (0.5 μg/ml; Sigma-Aldrich) in distilled water for 10 min at RT. After microscopic inspection for Hoechst fluorescence, cells were returned to sporulation medium. Only horsetail or pachytene cells that displayed Hoechst and healthy Zip1-GFP or Rec8-GFP fluorescence were recorded, making sure to compare equivalent stages. Z stacks (spaced 0.3 μm) of live cells were recoded with our 4D live cell microscope system (TILL [now FEI]) at RT. Image processing and voxel calculation was done using ImageJ and the Voxelcounter plugin (http://rsb.info.nih.gov/ij/plugins/voxel-counter.html). Cells that showed signs of cell death like nuclear hyper-condensation and increased auto-fluorescence were rarely encountered and excluded from analysis.

Since Hoechst staining is prone to artifacts due to dye flaring, we also measured the extension of Rec8-GFP expressing *S. pombe* horsetail nuclei and of Zip1-GFP expressing *S. cerevisiae* pachytene nuclei (see Fig. 6E) in live cell movies series and derived their nuclear volume. The volume of a horsetail nucleus was calculated by assuming that its extended shape reflects a cone sitting (tip = leading edge) on the base of a half sphere (trailing edge). Volume was thus calculated by adding the volume of a cone [Vc = 1/3 · r^2^ · π h], with h being the distance from the leading edge to the max diameter at the trailing end of the nucleus, to that of a half-sphere (r = ½ · max. diameter of the trailing end of the nucleus). V_ht_ = ((3/4 · π · r^3^)/2) + (1/3 · r^2^ · π h). The volume of a pachytene nucleus was calculated by obtaining a normalized diameter of a sphere d by summing widths (w) + height (h) divided by 2 [d = w + h/2] and calculating the nuclear volume assuming a spherical pachytene nucleus with V_p_ = 3/4 · π · r^3^. The mean and standard deviation was obtained from 24–31 GFP-positive meiocyte nuclei of each species.

### Statistics

For statistical evaluation data were compared using the t-test (http://www.graphpad.com). Data are shown as the mean ± standard deviation (SD) or as box plots unless otherwise indicated.

## Additional Information

**How to cite this article**: Illner, D. *et al.* Meiotic chromosome mobility in fission yeast is resistant to environmental stress. *Sci. Rep.* **6**, 24222; doi: 10.1038/srep24222 (2016).

## Supplementary Material

Supplementary Movie 1

Supplementary Information

## Figures and Tables

**Table 1:** Antioxidant gene expression in S. cerevisiae SK1.

Gene	expression (centile)	Gene	expression (centile)	Gene	expression (centile)	Gene	expression (centile)
CTT1	80	GRX8	80	MPD2	85	TRR1	95
GLR1	95	GSH2	95	PRX1	100	TRR2	90
GPX1	95	GTT1	95	RNR1	95	TRX1	100
GPX2	80	GTT2	80	RNR2	100	TRX2	100
GRX2	100	HYR1	100	RNR3	75	TSA1	100
GRX3	95	MET16	85	RNR4	100	TSA2	80
GRX6	95	MET3	80	SFA1	85	URE2	90
GRX7	95	MPD1	85	SOD1	100	YCF1	75

Genes involved in the antioxidant response are highly expressed during sporulation of the SK1 budding yeast strain. Data (4 h after meiosis induction) extracted from sgv.genouest.org.

**Table 2:** Strain list.

MS1428	*h^−^ GFP-atb2^+^::kanMX cut12^+^::CFP-natMX leu1 ura4*
UoA396^a^	*h^−smt0^ GFP-atb2^+^::natMX4 rec8^+^-GFP::kanMX6 uch2^+^-mCHERRY::ura4^+^ ade6-M210 ura4-D18*
UoA397^a^	*h^+S^ GFP-atb2^+^::natMX4 rec8^+^-GFP::kanMX6 uch2^+^-mCHERRY::ura4^+^ ade6-M216 ura4-D18*
UoA402^a^	*h^+S^/h^−smt0^ GFP-atb2^+^::natMX4/ GFP-atb2^+^::natMX4 rec8^+^-GFP::kanMX6/ rec8^+^-GFP::kanMX6 uch2^+^-mCHERRY::ura4^+^/uch2^+^-mCHERRY::ura4^+^ ade6-M210/ade6-M216 ura4-D18/ura4-D18*
UoA571	*h^+N^ lys1^+^::lacO his7^+^::lacI-GFP ade6-M216*
UoA572	*h^−^ lys1^+^::lacO his7^+^::lacI-GFP ade6-M210*
UoA579	*h^+N^/h^−^ lys1^+^::lacO/lys1^+^::lacO his7^+^::lacI-GFP/his7^+^::lacI-GFP ade6-M210/ade6-M216*
UoA575	*h^−^ his2::kanMX6-ura4^+^-lacO his7^+^::lacI-GFP ade6-M210 ura4-D18*
UoA576	*h^+N^ his2::kanMX6-ura4^+^-lacO his7^+^::lacI-GFP ade6-M216 ura4-D18*
UoA580	*h^+N^/h^−^ his2::kanMX6-ura4^+^-lacO/his2::kanMX6-ura4^+^-lacO his7^+^::lacI-GFP/his7^+^::lacI-GFP ade6-M210/ade6-M216 ura4-D18/ura4-D18*
FK1058^b^	*MAT a/α, ho::LYS2/ ho::LYS2 spo11::URA3/ spo11::URA3 ura3/ura3 ZIP1-GFP/ZIP1-GFP*
HW122^21^	*MATa/α lys2/lys2 ho::LYS2/ho::LYS2 ura3/ura3 ZIP1::GFP700/ ZIP1::GFP700*

^a^*GFP-atb2*^+^::*natMX4* strains are derivatives of MS1428 (FY17687) provided by the National BioResource Project (NBRP) of the MEXT, Japan.

^b^Kindly provided by Franz Klein, University of Vienna, Austria.
