# Supplementary material for: Meiotic chromosome mobility in fission yeast is resistant to environmental stress
Source: Sci Rep. 2016 Apr 14;6:24222. doi: 10.1038/srep24222 (PMC4831013; doi:10.1038/srep24222)
Supplement: Supplementary Information [file srep24222-s2.pdf]

# Meiotic chromosome mobility in fission yeast is resistant to environmental stress

Doris Illner [1], Alexander Lorenz [2], Harry Scherthan [1§]

[1] Institut für Radiobiologie der Bundeswehr in Verbindung mit der Univ. Ulm, Neuherbergstr. 11, D-80937 München, Germany
[2] Institute of Medical Sciences (IMS), University of Aberdeen, Foresterhill, Aberdeen AB25 2ZD, United Kingdom

[§] correspondence to HS: scherth@web.de

# Suppl. Fig. S1

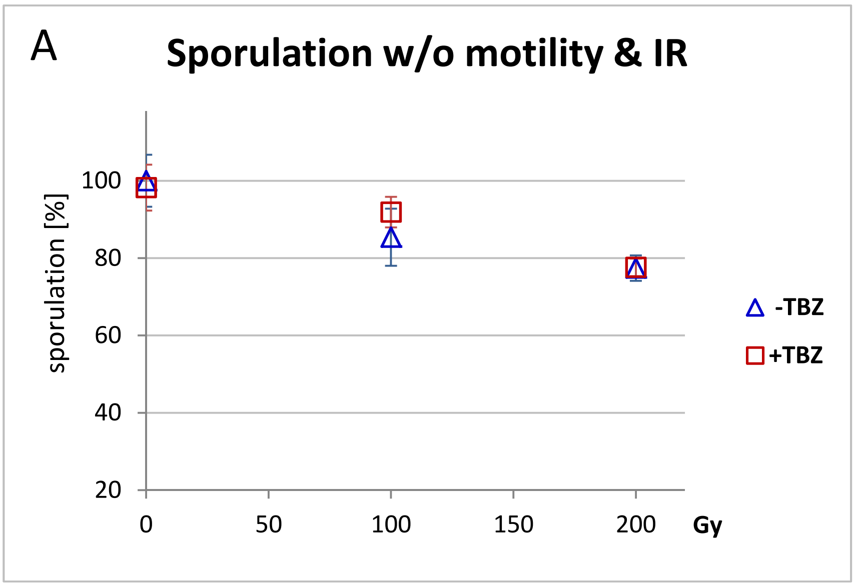


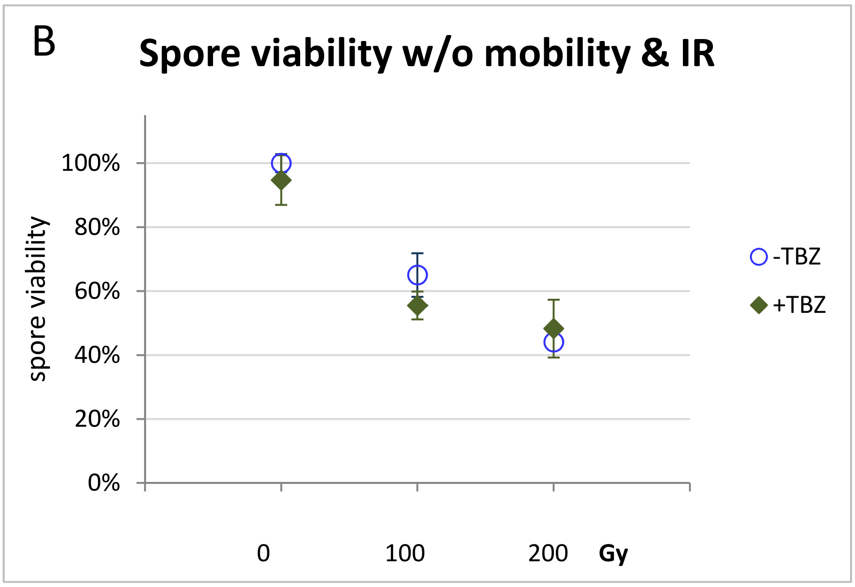


**Supplemental Fig. S1**: Sporulation (**A**) and spore viability (**B**) in *S. pombe* cells without (-TBZ) and with (+TBZ) microtubule inhibition of horsetail mobility by TBZ treatment (20µg/ml). Cells were irradiated with 100 and 200Gy X rays 3h after induction of sporulation. TBZ or DMSO (control) was added in equimolar amounts 30 min before IR. The observed values between the respective doses with (-TBZ) and without (+TBZ) horsetail mobility being similar (average of 6 technical repeats +-SD).

# Suppl. Fig. S2

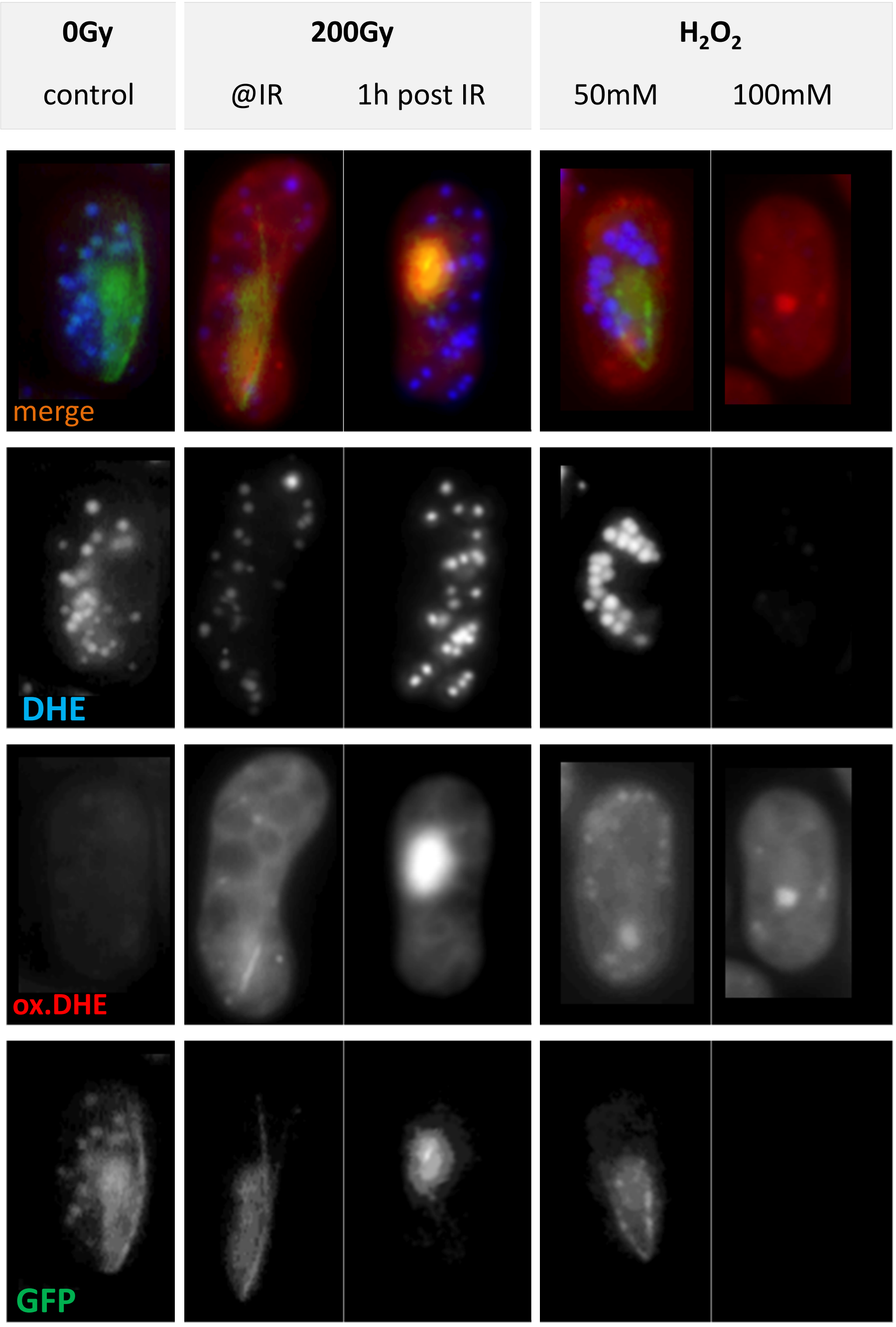


**Supplemental Fig. S2**: Split channel display of the images shown in Fig. 4A. Control horsetail cell (**0Gy**) with Rec8-GFP-labelled nucleus and MTs (green; GFP) showing blue accumulations of reduced DHE (DHE) in the cell. The DHE signals are also faintly seen in the green channel (GFP) due to bleed-through of the strong DHE fluorescence (DHE has a broad excitation spectrum between ~400-520nm and emits approx. 4% at 520nm used for FITC/GFP viewing [1]). **200Gy:** X irradiated cell displaying red oxidized DHE (ox.DHE) throughout the cell, while the nucleus and MT GFP fluorescence are faintly seen. Cell 1h post IR still showing slight red cytoplasmic ox.DHE labelling, while the nucleus appears orange due to colocalization of green Rec8-GFP and red Ethidium (ox.DHE) that stains DNA. MTs were too faint to be revealed by this exposure setting, while non-oxidized blue DHE aggregates are still seen. **$H_2O_2$:** Cells treated with 50mM $H_2O_2$ display red cytoplasmic ox.DHE and a green horsetail nucleus; 100mM $H_2O_2$ completely oxidized all DHE to red ox.DHE and quenched GFP fluorescence.

[1] https://www.thermofisher.com/de/de/home/life-science/cell-analysis/labeling-chemistry/fluorescence-spectraviewer.html?ICID=svtool&UID=2OHEtDNA

# Suppl. Fig. S3

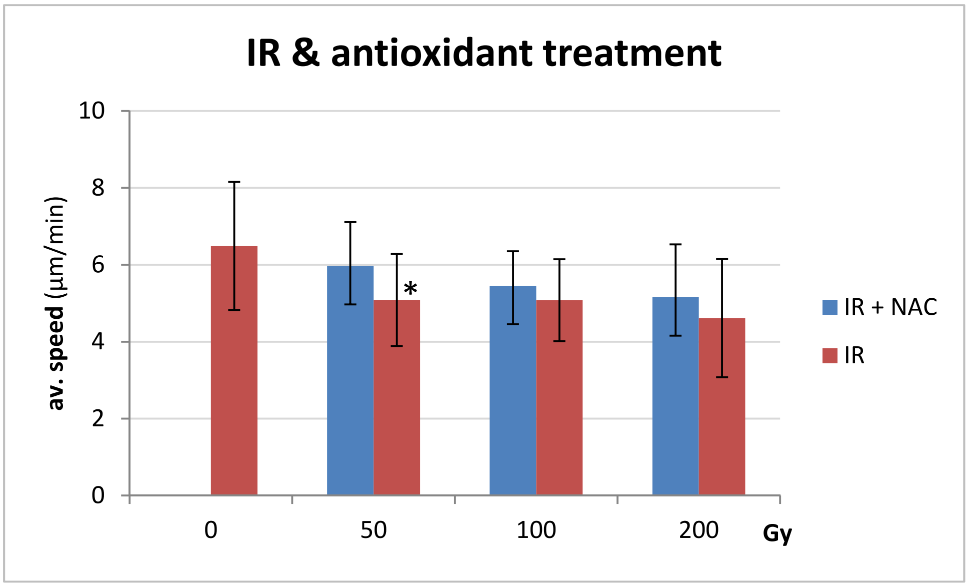


**Supplemental Fig. S3**: Antioxidant protection of horsetail mobility. Irradiation of horsetail cells with 50 – 200Gy leads to a reduction of the average horsetail speed in the cells without antioxidant protection. The presence of the antioxidant NAC (10mM) partially rescued horsetail mobility in irradiated live cells. Average speed (µm/min) and SD shown. The difference between 50Gy and 50Gy+NAC being significant (*; p=0.005), while at higher doses the differences are insignificant.

**Suppl. Video 1**

Time lapse cinematography of Rec8-GFP and tubulin-GFP expressing *S. pombe* horsetail-stage cells showing the typical mobility of meiotic nuclei driven by astral MT oscillations. Frame rate 0.33Hz, 100x magnification.
